# Impaired natural killer cell subset phenotypes in human obesity

**DOI:** 10.1007/s12026-018-8989-4

**Published:** 2018-03-20

**Authors:** Ina Bähr, Janine Jahn, Alexander Zipprich, Inge Pahlow, Julia Spielmann, Heike Kielstein

**Affiliations:** 1 Department of Anatomy and Cell Biology, Faculty of Medicine, Martin Luther University Halle-Wittenberg, Grosse Steinstrasse 52, 06108 Halle/Saale, Germany; 2 Clinic of Internal Medicine I, University Hospital of Martin Luther University Halle-Wittenberg, Ernst-Grube-Strasse 40, 06120 Halle/Saale, Germany

**Keywords:** Human natural killer cells, Immune cell functions, Adipocytokines, Obesity

## Abstract

Obesity is associated with alterations in functionality of immune cells, like macrophages and natural killer (NK) cells, leading to an increased risk for severe infections and several cancer types. This study aimed to examine immune cell populations and functional NK cell parameters focusing on NK cell subset phenotypes in normal-weight and obese humans. Therefore, peripheral blood mononuclear cells (PBMCs) were isolated from normal-weight and obese individuals and analyzed by flow cytometry. Results show no significant changes in the frequency of monocytes, B lymphocytes, or NKT cells but a significantly increased frequency of T lymphocytes in obesity. The frequency of total NK cells was unaltered, whereas the number of low cytotoxic CD56^bright^ NK cell subset was increased, and the number of high cytotoxic CD56^dim^ NK cell subset was decreased in obese subjects. In addition, the frequency of CD56^bright^ NK cells expressing the activating NK cell receptor NKG2D as well as intracellular interferon (IFN)-γ was elevated in the obese study group. In contrast, the frequency of NKG2D- and IFN-γ-positive CD56^dim^ NK cells was lower in obesity compared to normal-weight individuals. Moreover, the expression of the activation marker CD69 was decreased in NK cells, which can be attributed to a reduction of CD69-positive CD56^dim^ NK cells in obese subjects. In conclusion, data reveal an impaired NK cell phenotype and NK cell subset alterations in obese individuals. This NK cell dysfunction might be one link to the higher cancer risk and the elevated susceptibility for viral infections in obesity.

## Introduction

The rising prevalence of overweight and obesity has become a major global health challenge as excess body weight is associated with nearly 4 million deaths and 120 million disability-adjusted life-years [1]. Obesity is a major risk factor for several non-communicable chronic diseases, like type 2 diabetes mellitus [2], cardiovascular diseases [3], and musculoskeletal [4] and kidney disorders [5], as well as several cancer types [6]. In addition, the susceptibility to infections as well as the infection-associated mortality is increased in obese individuals [7–9]. Obesity is associated with metaflammation, a state of a systemic chronic low-grade inflammation, associated with dysfunctions of several immune cells, like B lymphocytes, T lymphocytes, and macrophages [10–12]. Moreover, previous studies demonstrated an impaired functionality of natural killer (NK) cells in the state of obesity [13–17].

NK cells are effector lymphocytes of the innate immune system rapidly killing virus-infected and tumor cells without prior sensitization while remaining tolerant of normal cells. Natural killer cells express both activating and inhibitory receptors that are involved in regulating NK cell effector functions. NK cell-mediated target cell lysis is induced via exocytosis of granzymes and perforin. Besides direct cytotoxic effects, NK cells secrete several cytokines, like tumor necrosis factor (TNF)-α and interferon (IFN)-γ, in order to regulate the adaptive immune response [18].

Human NK cells are usually defined as CD3^−^/CD56^+^ large granular lymphocytes that can be subcategorized based on their CD56 (neural cell adhesion molecule; NCAM) expression level. The low-density (CD56^dim^) NK cell subset is the numerically major NK cell subpopulation in peripheral blood and has been described as the more cytotoxic subset with a high potential to produce granzymes and perforin. In contrast, high-density (CD56^bright^) NK cells are less cytotoxic but mediate immunoregulatory effects by secreting large amounts of cytokines [19, 20].

Several studies investigated NK cell functionality in obesity. In vitro analyses revealed evidence for an altered cytotoxicity, cytokine secretion, and phenotype of primary NK cells and NK cell lines after incubation with the adipocytokines leptin or adiponectin as well as adipocytokine containing supernatants derived from cultured adipocytes [21–27].

Results of animal studies demonstrated inhibitory effects of high-fat feeding on NK cell-mediated target cell lysis [9, 28–31]. Interestingly, the obesity-associated NK cell dysfunction could be ameliorated by transfer of NK cells from obese rats in normal-weight rats, indicating that NK cell functionality depends on the surrounding metabolic environment [32]. In humans, data on functionality and phenotype of peripheral NK cells in obesity are limited and partially conflicting. For instance, inconsistent data exist about the effect of obesity on peripheral NK cell number [13, 15, 33–37]. Moreover, results of previous studies demonstrated heterogeneous effects of obesity on receptor expression or IFN-γ secretion of NK cells [13, 33–35]. In contrast, degranulation capacity and cytotoxicity against tumor cells were consistently found to be decreased in obese subjects compared to normal-weight individuals [13, 15]. Until now, no investigations exits investigating NK cell parameters specifically in the cytotoxic CD56^dim^ and the immunomodulatory CD56^bright^ NK cell subpopulations. Therefore, the aim of this study was to analyze immune cell populations and functional NK cell parameters with the focus on NK cell subset-specific differences in normal-weight and obese humans.

## Material and methods

### Study population

The study was approved by the ethics committee of the Faculty of Medicine, Martin Luther University Halle-Wittenberg, Halle/Saale, Germany. Each subject signed an informed declaration of consent. Exclusion criteria were chronic infections, acute diseases, severe endocrine disorders (e.g., severe hypo- or hyperthyroidism), immunosuppressive therapy, and known malignant tumors. In addition, woman and male subjects of an age less than 50 years or aged over 70 years were excluded. Based on their body mass index (BMI), subjects were classified in a normal-weight study group with BMI < 25 kg/m^2^ (*n* = 6) and an obese study group with BMI > 30 kg/m^2^ (*n* = 8). All subjects were patients of the Clinic for Internal Medicine I, University Hospital of Martin Luther University Halle-Wittenberg, Halle/Saale, Germany, treated in an outpatient setting.

### Blood collection and analyses of plasma adipocytokines

Blood samples from all subjects were collected in monovettes prepared with the anticoagulant ethylene-diamine-tetra-acetic acid (EDTA). Plasma samples were obtained by centrifugation and stored at − 80 °C until analyzed.

Measurements of concentrations of the adipocytokines resistin, adiponectin, leptin, interleukin (IL)-6, IL-1β, and TNF-α in plasma samples were performed using a multiplex immunoassay (Merck Millipore, Darmstadt, Germany) following the manufacturer’s protocol. In brief, standards and samples were diluted in an equal volume of assay puffer and incubated with antibody-immobilized beads at 4 °C for 16 h. After washing, a biotinylated detection antibody was added for 1 h followed by incubation with streptavidin-phycoerythrin for 30 min at room temperature. Adipocytokine levels were determined using the LiquiChip luminex 200 system (Qiagen, Hilden, Germany).

### Isolation of PBMCs

Isolation of peripheral blood mononuclear cells (PBMCs) was performed immediately after blood sampling. Blood was diluted with phosphate-buffered saline (PBS), and PBMCs were separated by density gradient centrifugation using biocoll separation solution (Biochrom AG, Berlin, Germany). After collection of PBMCs from the interphase and washing with PBS, cell number was determined.

### Flow cytometric analyses

For flow cytometric analyses, PBMCs were stained with the following antibodies (all from BD Biosciences, San Diego, USA, unless otherwise indicated): CD3 conjugated with phycoerythrin (PE)-Cy 7, CD4 conjugated with allophycocyanin (APC), CD 8 conjugated with PE, CD14 conjugated with fluorescein isothiocyanate (FITC), CD20 conjugated with APC-H7, CD56 conjugated with APC, CD253 (TRAIL; tumor necrosis factor-related apoptosis-inducing ligand) conjugated with PE, Ob-R (leptin receptor) conjugated with fluorescein (R&D Systems, Minneapolis, MN, USA), CD314 (NKG2D) conjugated with PE-CF594, CD25 conjugated with PE CF594, CD69 conjugated with FITC, and CD107a conjugated with PE. In addition, matched isotype control antibodies were used for TRAIL and Ob-R. PBMCs were incubated with the antibodies for 30 min at 4 °C protected from light.

For intracellular staining, cell surface staining was performed by incubation with the CD56 APC antibody and the CD3 PE-Cy7 antibody for 30 min at 4 °C. Subsequently, PBMCs were fixed in 4% paraformaldehyde for 10 min at 4 °C. After washing, cells were incubated with the IFN-γ antibody conjugated with PE-C7 and the granzyme A antibody conjugated with FITC (both from BD Biosciences) or matched isotype control antibodies, diluted in saponin buffer (0.1% saponin and 0.01 M hydroxyethyl piperazineethanesulfonic acid; HEPES) for 30 min at 4 °C. After washing and suspending in measuring puffer (PBS supplemented with 0.1% bovine serum albumin and 0.1% sodium azide), all samples were analyzed by flow cytometry using the LSR Fortessa (BD Biosciences) and the FACSDiva software version 6.2 (BD Biosciences).

### Statistical analyses

Statistical analyses were performed using an unpaired *t* test to compare results between the two study groups. Pearson’s correlation test was used to investigate the association between different parameters related to the BMI of all subjects of the study. All data analyses were performed using the GraphPad Prism 7 software (GraphPad Software, La Jolla, USA). Differences were considered significant if *P* values were less than 0.05. Data are represented as means ± standard error of the mean (SEM).

## Results

### Study population

The study subjects were aged between 51 and 68 years. No significant differences in age and height were observed between the normal-weight and obese study group (Table 1). Obese individuals showed a significantly higher body weight and BMI compared to the normal-weight study group (Table 1).

**Table 1:** Study population

	Normal weight (*n* = 6)	Obese (*n* = 8)	*P* value
Age (years)	59.2 ± 2.8	59.5 ± 2.0	0.921
Height (m)	1.8 ± 0.03	1.8 ± 0.02	0.389
Weight (kg)	73.8 ± 3.7	104.4 ± 3.3	< 0.0001***
BMI (kg/m^2^)	22.5 ± 0.8	33.0 ± 0.8	< 0.0001***

Data are expressed as mean ± standard error of the mean (SEM)

*BMI* body mass index

***Significant differences (*P* < 0.0001)

### Plasma adipocytokine concentrations

A multiplex immunoassay was performed to analyze plasma concentrations of obesity-related parameters. Results showed significantly higher plasma leptin concentrations in obese individuals compared to normal-weight subjects (Table 2). No significant differences were detected in plasma concentrations of the adipocytokines adiponectin, IL-1β, IL-6, resistin, and TNF-α (Table 2).

**Table 2:** Plasma adipocytokine concentrations

	Normal weight (*n* = 6)	Obese (*n* = 8)	*P* value
Adiponectin (μg/ml)	12.4 ± 0.4	11.1 ± 0.9	0.2385
IL-1β (pg/ml)	21.2 ± 1.1	22.1 ± 1.2	0.6095
IL-6 (pg/ml)	23.1 ± 3.5	25.5 ± 2.8	0.6084
Leptin (ng/ml)	3.3 ± 1.0	16.5 ± 4.8	0.0478*
Resistin (ng/ml)	12.5 ± 1.1	17.7 ± 0.6	0.8925
TNF-α (pg/ml)	51.4 ± 4.7	48.3 ± 1.8	0.5251

Data are expressed as mean ± standard error of the mean (SEM)

*IL* interleukin, *TNF-α* tumor necrosis factor α

*Significant differences (*P* < 0.05)

### Analyses of blood leucocyte subsets

To investigate blood leucocyte subsets in normal-weight and obese individuals, FACS analyses were performed with isolated PBMCs. The frequency of monocytes, B lymphocytes, and NKT cells did not differ between the two study groups (Table 3). In contrast, the frequency of total T lymphocytes was significantly decreased in obese individuals compared to the normal-weight study group. Analyses of T cell subsets showed that the frequency of T helper cells (CD4+) was not altered in obesity, and the frequency of cytotoxic T cells (CD8+) was slightly, but not significantly, reduced in the obese study group (Table 3).

**Table 3:** Immune cell populations

	Normal weight (*n* = 6)	Obese(*n* = 8)	*P* value
Monocytes (CD14^+^; % of PBMCs)	13.8 ± 1.0	18.0 ± 2.5	0.2175
B lymphocytes (CD20^+^; % of PBMCs)	7.5 ± 1.2	9.4 ± 1.7	0.4367
T lymphocytes (CD3^+^; % of PBMCs)	68.3 ± 3.6	54.4 ± 3.4	0.0145*
T helper cells (CD3^+^, CD4^+^; % of T lymphocytes)	65.1 ± 4.8	68.9 ± 2.5	0.4786
Cytotoxic T cells (CD3^+^, CD8^+^; % of T lymphocytes)	24.5 ± 3.8	17.4 ± 3.1	0.1748
Natural killer T cells (CD3^+^, CD56^+^; % of PBMCs)	3.9 ± 0.9	3.8 ± 1.1	0.9769

Data are expressed as mean ± standard error of the mean (SEM)

*PBMCs* peripheral blood mononuclear cells

*Significant differences (*P* < 0.05)

### Investigations on NK cells and NK cell subsets

**Fig. 1:**
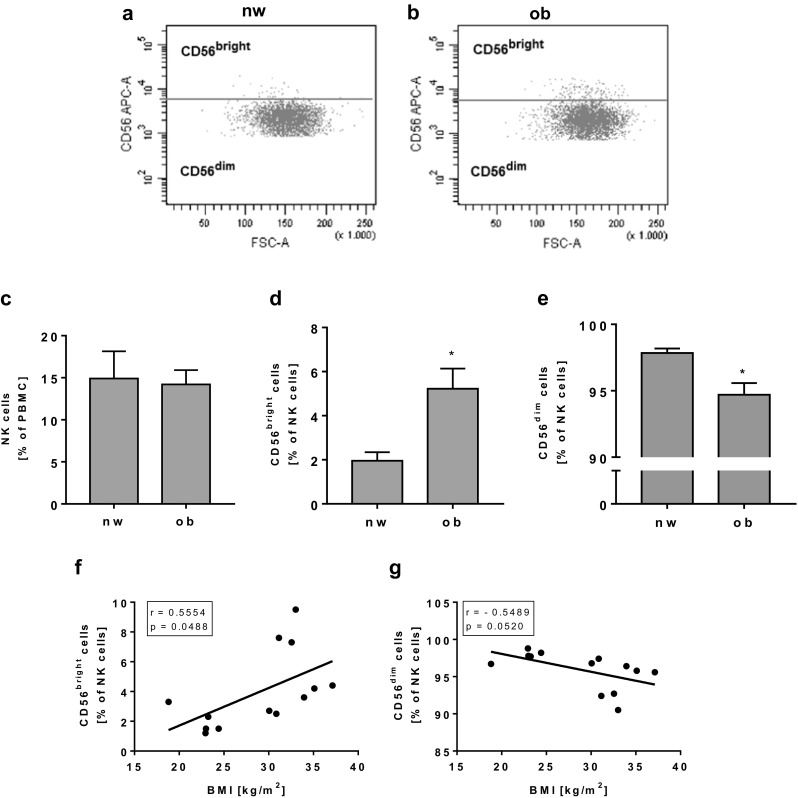
Flow cytometric analyses of NK cells and NK cell subsets in PBMCs (peripheral blood mononuclear cells) isolated from normal-weight (nw) and obese (ob) individuals. **a**, **b** Exemplary FACS plots of CD56^bright^ and CD56^dim^ NK cells of a normal-weight and an obese subject. **c** Frequency of NK cells in PBMCs. **d**, **e** Expression of CD56^bright^ (**d**) and CD56^dim^ (**e**) NK cells. Data are expressed as mean ± SEM. *P < 0.05 compared to the normal-weight study group. **f**, **g** Correlation of the percentage of CD56^bright^ (**f**) and CD56^dim^ (**g**) NK cells with the individual BMI (body mass index) of each subject

As demonstrated in Fig. 1, FACS analyses showed no significant differences in the overall frequency of NK cells comparing normal-weight and obese individuals (Fig. 1a–c). NK cells were subsequently separated into CD56^bright^ or CD56^dim^ subset based on the expression level of CD56. Interestingly, the percentage of CD56^bright^ NK cells was significantly increased and the percentage of CD56^dim^ NK cells was significantly decreased in obesity (Fig. 1d, e). No significant effect was observed correlating the NK cell frequency with the individual BMI of each subject (data not shown). The correlation of the BMI with the expression of CD56^bright^ or CD56^dim^ NK cells of all normal-weight and obese individuals resulted in a significant positive correlation between CD56^bright^ NK cells and BMI as well as a tendentially negative correlation between CD56^dim^ NK cells and BMI (Fig. 1f, g).

### Analyses of NKG2D receptor expression on NK cells

**Fig. 2:**
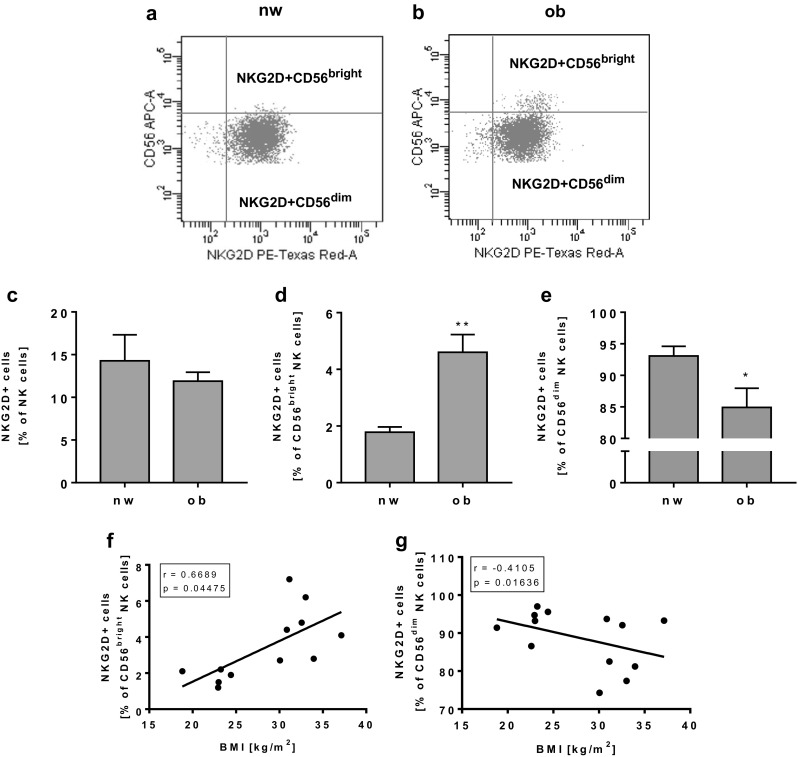
Flow cytometric analyses of NKG2D receptor expression on NK cells and NK cell subsets in PBMCs isolated from normal-weight (nw) and obese (ob) individuals. **a**, **b** Exemplary FACS plots of NKG2D expression in CD56^bright^ and CD56^dim^ NK cells of a normal-weight and an obese subject. **c** Frequency of NKG2D-positive NK cells in PBMCs. **d**, **e** Expression of NKG2D in CD56^bright^ (**d**) and CD56^dim^ (**e**) NK cells. Data are expressed as mean ± SEM. *P < 0.05; **P < 0.01 compared to the normal-weight study group. **f**, **g** Correlation of the percentage of NKG2D expressing CD56^bright^ (**f**) and CD56^dim^ (**g**) NK cells with the individual BMI of each subject

Analyses of activating NK cell receptor expression revealed no changes in NKG2D receptor expression in total NK cells (Fig. 2c) and no correlation between NKG2D receptor expression in total NK cells and BMI of all subjects (data not shown). In contrast, NKG2D expression was significantly increased in CD56^bright^ NK cells and significantly decreased in CD56^dim^ NK cells in obese subjects (Fig. 2d, e). Correlating the BMI with the NKG2D receptor expression on CD56^bright^ or CD56^dim^ NK cells of all normal-weight and obese individuals showed a significant positive correlation between NKG2D-expressing CD56^bright^ NK cells and BMI and a significantly negative correlation between NKG2D-expressing CD56^dim^ NK cells and BMI (Fig. 2f, g).

### Analyses of CD69 receptor expression on NK cells

**Fig. 3:**
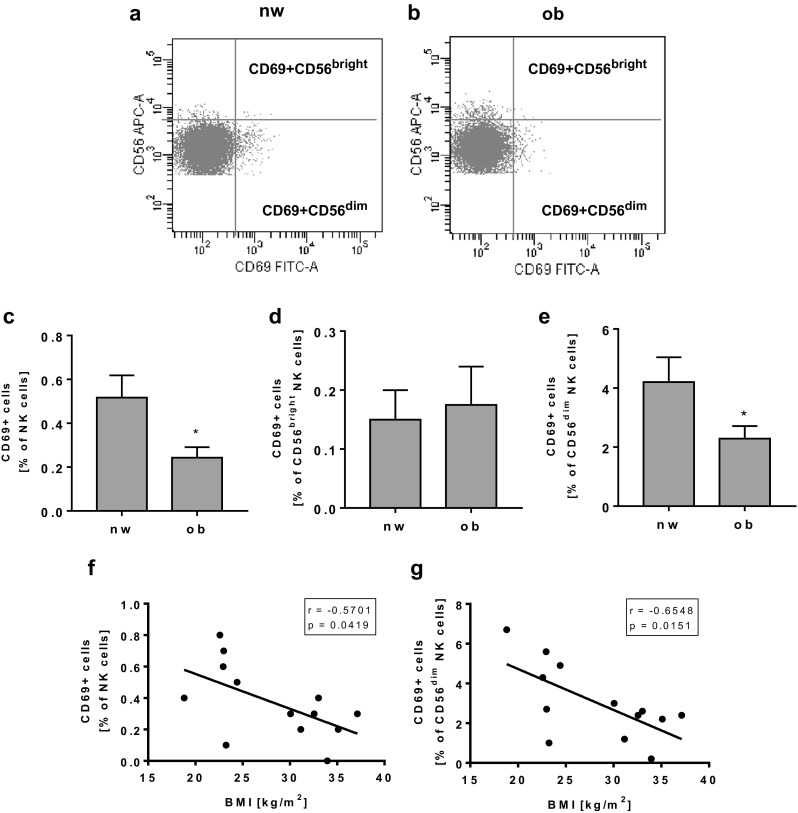
Flow cytometric analyses of CD69 expression on NK cells and NK cell subsets in PBMCs isolated from normal-weight (nw) and obese (ob) individuals. **a**, **b** Exemplary FACS plots of CD69 expression in CD56^bright^ and CD56^dim^ NK cells of a normal-weight and an obese subject. **c** Frequency of CD69-positive NK cells in PBMCs. **d**, **e** Expression of CD69 in CD56^bright^ (**d**) and CD56^dim^ (**e**) NK cells. Data are expressed as mean ± SEM. *P < 0.05 compared to the normal-weight study group. **f**, **g** Correlation of the percentage of CD69-expressing total NK cells (**f**) and CD56^dim^ NK cells (**g**) with the individual BMI of each subject

Expression of the activating NK cell receptor CD69 in total NK cells as well as in CD56^dim^ NK cells was significantly lower in obese subjects compared to normal-weight subjects (Fig. 3c, e). The frequency of CD69-expressing CD56^bright^ NK cells showed no differences between normal=weight and obese individuals (Fig. 3d). Moreover, the CD69 expression in total NK cells and CD56^dim^ NK cells was negatively correlated with the BMI of all study subjects (Fig. 3f, g), whereas no correlation between CD69 expression between CD56^bright^ NK cells and BMI was observed (data not shown).

### Expression of other NK cell surface receptors

**Fig. 4:**
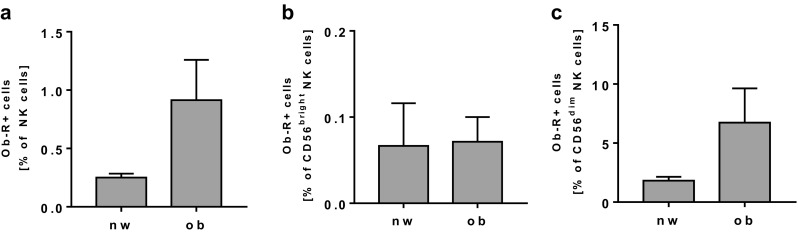
Flow cytometric analyses of leptin receptor (Ob-R) expression in NK cells and NK cell subsets in PBMCs isolated from normal-weight (nw) and obese (ob) individuals. **a** Frequency of Ob-R expressing NK cells in PBMCs. **b**, **c** Expression of Ob-R in CD56^bright^ (**b**) and CD56^dim^ (**c**) NK cells. Data are expressed as mean ± SEM

No significant differences in the expression levels of TRAIL, CD25, and CD107a in total NK cells as well as in CD56^bright^ or CD56^dim^ NK cells were observed comparing the two study groups (data not shown). Expression of the leptin receptor was increased, without reaching the significance level in total NK cells as well as in CD56^dim^ NK cells, whereas no difference in leptin receptor expression was observed in CD56^bright^ NK cells (Fig. 4a–c).

### Analyses of intracellular NK cell markers

**Fig. 5:**
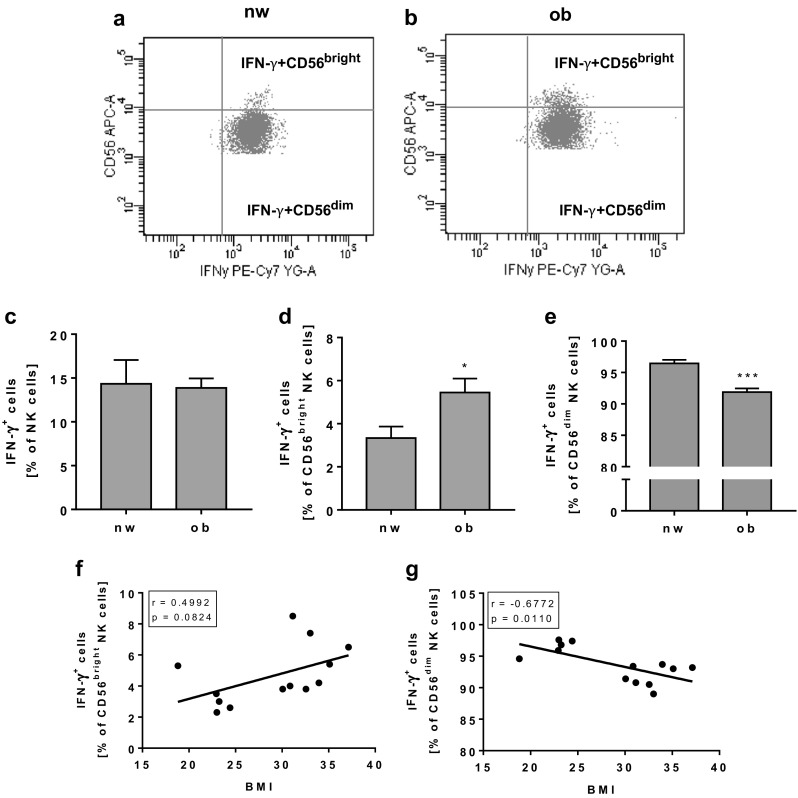
Flow cytometric analyses of interferon gamma (IFN-γ) expression on NK cells and NK cell subsets in PBMCs isolated from normal-weight (nw) and obese (ob) individuals. **a**, **b** Exemplary FACS plots of IFN-γ expression in CD56^bright^ and CD56^dim^ NK cells of a normal-weight and an obese individual. **c** Frequency of IFN-γ-expressing NK cells in PBMCs. **d**, **e** IFN-γ expression in CD56^bright^ (**d**) and CD56^dim^ (**e**) NK cells. Data are expressed as mean ± SEM. *P < 0.05; ***P < 0.001 compared to the normal-weight study group. **f**, **g** Correlation of the percentage of IFN-γ-expressing CD56^bright^ NK cells (**f**) and CD56^dim^ (**g**) NK cells with the individual BMI of each subject

FACS analyses of the serine protease granzyme A revealed no significant differences in the frequency of total NK cells as well as in CD56^bright^ or CD56^dim^ NK cell subsets (data not shown). Moreover, the frequency of IFN-γ expressing total NK cells was not altered in obesity (Fig. 5c). In contrast, the percentage of IFN-γ-expressing CD56^bright^ NK cells was significantly increased and the percentage of CD56^dim^ NK cells was significantly decreased in the obese study group (Fig. 5d, e). Correlation analyses of BMI and the frequency of total NK cells showed no significant effects (data not shown). Correlating the BMI with the IFN-γ expression in CD56^bright^ or CD56^dim^ NK cells of all normal-weight and obese individuals showed a tendentially positive correlation between IFN-γ-expressing CD56^bright^ NK cells and BMI and a significant negative correlation between IFN-γ-expressing CD56^dim^ NK cells and BMI (Fig. 5f, g).

## Discussion

Numerous investigations exist analyzing the influence of obesity on NK cells in animals showing an altered phenotype and functionality of NK cell in obese rats and mice [9, 14, 28–32]. In contrast, studies analyzing NK cell characteristics in human individuals, especially focusing on NK cell subsets in overweight and obesity, are rare.

Results of this study investigating normal-weight compared to obese individuals demonstrate that obesity has no influence on the frequency of total NK cells, whereas the CD56^dim^/CD56^bright^ ratio was decreased in obese subjects. Previous investigations provided conflicting results about the number of total NK cells comparing normal-weight and obese individuals but did not differentiate between CD56^bright^ and CD56^dim^ NK cells subsets [13, 15, 33, 35, 37]. The lower CD56^dim^/CD56^bright^ ratio in obese subjects may be one cause for the impaired lytic activity of NK cells against virus-infected and tumor cells observed by several studies [9, 15] and therefore contribute to the higher cancer risk and susceptibility for infections under obese conditions. In addition, an increase of the immunoregulatory CD56^bright^ NK cell number may lead to an enhanced secretion of cytokines, like TNF-α and IFN-γ, and therefore potentially contribute to the obesity-induced low-grade inflammation state.

Explanations for the shift of the CD56^dim^/CD56^bright^ ratio in obesity remain unclear. Two models of NK cell subset development have been proposed. On the one hand, convincing data exist demonstrating that both NK cell subsets develop from a common NK cell precursor and subsets differentiate into CD56^bright^ or CD56^dim^ cells in dependence of the microenvironment [38]. On the other hand, data indicate that CD56^bright^ cells are immature precursors of the finally differentiated CD56^dim^ NK cells [39]. Interestingly, studies observed that CD56^dim^ NK cells can also convert to a CD56^bright^ NK cell phenotype under the influence of specific receptor ligands and cytokines [40, 41]. In addition, sex steroid hormones were shown to reduce the CD56^dim^/CD56^bright^ ratio in endometrial tissue [42]. Therefore, it can be assumed that the observed shift of the CD56^dim^/CD56^bright^ ratio in obese humans may be caused by a conversion of CD56^dim^ in CD56^bright^ NK cells, possibly induced by obesity-related metabolites. Previous in vitro investigations of Huebner et al. could not find any effects of an incubation of human NK cells with an adipocyte-conditioned medium on the NK cell subset distribution [23]. Thus, further studies investigating the influence of different single adipocytokines on interconversions of NK cell subpopulations are required to specify the influence of obesity-associated metabolites on NK cell phenotype and functionality.

The NKG2D receptor is an activating receptor triggering the cytotoxic activity and the cytokine secretion of NK cells [38, 39]. Previous studies on human NK cells demonstrated that NKG2D receptor expression can be decreased by leptin incubation [23]. In addition, diet-induced obesity in rats led to a decreased NKG2D mRNA expression in splenic tissue accompanied with higher metastasis and tumor development [23, 40]. In contrast, in humans, the frequency of NKG2D-expressing NK cells in blood as well as the number of NKG2D-positive NK cells in adipose tissue was higher in obese subjects compared to normal-weight subjects [16, 41]. In the present study, the frequency of total NK cells expressing the NKG2D receptor did not differ between normal-weight and obese subjects. Interestingly, the results show an increase in NKG2D-expressing CD56^bright^ NK cells and a decrease in NKG2D-positive CD56^dim^ NK cells.

In addition to the activating NK cell receptor NKG2D, we investigated the CD69 expression in peripheral NK cells of normal-weight and obese subjects. CD69 was described as an early activation marker of NK cells. An increase of CD69 expression leads to a higher cytotoxic capacity of NK cells [43, 44]. Previous investigations demonstrated an increased CD69 expression in NK cells in obesity [13, 33, 37]. In contrast, a significantly decreased CD69 expression in NK cells of obese subjects was determined in the present study which could primarily be attributed to a reduced CD69 expression in the cytotoxic CD56^dim^ NK cell subset.

These data lead to the assumption that the impaired cytotoxicity of NK cells against target cells in obesity may be caused by a lower expression of NKG2D and CD69 in the cytotoxic CD56^dim^ NK cell subset. As previous investigations demonstrated reduced expression levels of activating NK cell receptors in obese rats, the present study could confirm these results in NK cells of normal-weight and obese humans [25, 45]. CD56^dim^ NK cells represent at least 90% of all peripheral blood NK cells [19]. Therefore, it can be assumed that a decreased expression level of activating NK cell receptors on the CD56^dim^ NK cell subset may lead to a substantial reduction of NK cell functionality. In the future, cytotoxicity assays with NK cells isolated from normal-weight and obese individuals could be performed and correlated with expression levels of CD56^dim^ and CD56^bright^ NK cells to concretize the functional importance of a shift of NK cell subpopulations.

Similarly to NKG2D expression, obese individuals of the present study revealed an increased frequency of IFN-γ-expressing CD56^bright^ NK cells and a decrease in IFN-γ-positive CD56^dim^ NK cells, whereas no differences in IFN-γ expressing total NK cells were observed comparing normal-weight to obese subjects. These results indicate that the cytokine-mediated immunoregulatory effect on CD56^bright^ NK cell subset is upregulated in obesity. This might contribute to functional disturbances of other immune cells like the well-described activation and polarization of monocytes towards the proinflammatory M1 type macrophages in obesity [46]. Interestingly, in line with other studies on human NK cells, we observed a higher amount of IFN-γ expressing CD56^dim^ NK cells compared to IFN-γ expression in CD56^bright^ NK cells [47]. These results are at variance with the general consideration that CD56^bright^ NK cells are the major source of cytokines [19, 20]. Previous investigations on cytokine secretion of NK cell subsets demonstrated a rapid and abundant IFN-γ production of CD56^dim^ NK cells upon target cell recognition, whereas CD56^bright^ NK cells were described to guarantee a later but more prolonged cytokine production [48, 49]. As these data indicate a prominent role of CD56^dim^ NK cells for cytokine secretion, future studies are required to revise and specify the functional capacities of the CD56^dim^ and CD56^bright^ NK cell subsets [50].

In contrast to other studies, we observed no differences in granzyme A or CD107a expression on NK cells comparing normal-weight and obese subjects [13]. These discrepancies may occur since we analyzed basal expression levels of unstimulated NK cells. Further investigations analyzing degranulation and granzyme or perforin secretion after prior NK cell stimulation with interleukins or tumor cells could be helpful to detect possible obesity-associated effects on these functional NK cell parameters.

Obesity is associated with chronically increased plasma leptin concentrations [51, 52]. Leptin is known to mediate several regulatory effects on the adaptive and innate immune system [53]. Recent investigations revealed evidence that rodent and human NK cells express leptin receptors [14, 24, 35, 54]. In line with data of previous analyses in rats and humans, results of this study demonstrate that the leptin receptor expression on peripheral NK cells is tendentially increased in obese subjects [14, 35]. Former in vitro and in vivo studies showed that leptin has a diminishing effect on cytotoxicity and cytokine secretion of NK cells and modulates NK cell surface receptor expression [14, 24, 25, 35]. The discrepancy between the increased leptin receptor expression in obesity and the altered functionality in NK cells after leptin incubation could be explained by an impaired post-receptor leptin signaling cascade in NK cells [14, 24, 27, 35]. Besides leptin, other obesity-related metabolites, like adiponectin or IL-6, are shown to influence NK cell functionality [15, 21, 22, 27, 55]. In the present study, only plasma leptin levels were enhanced in the obese study group, whereas concentrations of other plasma adipocytokines did not differ between normal-weight and obese subjects. These results does not point to a proinflammatory state in the obese subjects but indicate that high leptin concentrations may lead to the altered NK cell phenotype observed in obese subjects.

Obesity is accompanied with dysfunctions and altered frequencies of different immune cells [10–12, 25]. Results of the present study demonstrate a significant decrease of the frequency of T lymphocytes in obese subjects compared to normal-weight subjects. In accordance, previous data had already shown a decreased number of total T lymphocytes in peripheral blood of obese individuals [56]. Interestingly, body weight reduction by restriction of energy intake or physical activity resulted in an increase of T cell number in obese humans [47, 56]. Beside the impact of obesity on alterations of NK cell phenotype and functionality, obesity-induced changes of T cell number and function may be one cause for the higher susceptibility for viral infections and cancer diseases in obesity.

It has been demonstrated that a reduction of body weight and fat mass after exercise and dietary intervention or bariatric surgery lead to an improved NK cell functionality in obese individuals [47, 57–59]. In addition, animal experiments revealed evidence for an increased NK cell infiltration and an upregulation of NK cell-relevant immune cell markers in tumor tissue associated with a reduced tumor growth after voluntary exercise training [60]. These data underline the highly preventive effect of physical exercise as well as body weight and fat mass reduction in obesity. Until now, data about the influence of weight reduction or regular physical activity on the distribution of NK cell subsets are limited. In a lifestyle intervention study of our working group with obese volunteers, body weight reduction had no effect on the distribution of CD56^bright^ and CD56^dim^ NK cells. Interestingly, results of this study showed a significantly increased frequency of IFN-γ-positive CD56^dim^ NK cells after body weight reduction, whereas IFN-γ-positive CD56^bright^ NK cells were unchanged [47]. In accordance with the results of this study, these data indicate that NK cell subsets were differentially regulated in dependence of body mass index in humans. Previous investigations already demonstrated that the impaired NK cell function in obesity is associated with an increased tumor development in rats and mice [25, 45, 59]. Therefore, future investigations about the influence of body weight loss on the functionality and phenotype of NK cell subsets could concretize the tumor-preventative effect of fat mass reduction in obese individuals.

In conclusion, results of this study demonstrated for the first time an altered NK cell phenotype and a shift in NK cell subpopulations in obese individuals. These impaired NK cell characteristics may contribute to the higher susceptibility for viral infections and the increased cancer risk in obesity.
